# Adult attachment style and cortisol responses in women in late pregnancy

**DOI:** 10.1186/s40359-016-0105-8

**Published:** 2016-01-11

**Authors:** José Manuel Costa-Martins, Mariana Moura-Ramos, Maria João Cascais, Carlos Fernandes da Silva, Henriqueta Costa-Martins, Marco Pereira, Rui Coelho, Jorge Tavares

**Affiliations:** Department of Anaesthesiology, Maternity Hospital Alfredo da Costa, Rua Viriato, 1069-089 Lisbon, Portugal; Faculty of Psychology and Educational Sciences, University of Coimbra, Rua do Colégio Novo, 3001-802 Coimbra, Portugal; Clinical Pathology, Biochemistry, Maternity Hospital Alfredo da Costa, Rua Viriato, 1069-089 Lisbon, Portugal; Education Department, University of Aveiro, Campus de Santiago, 3810-193 Aveiro, Portugal; ISPA, University Institute, Rua Jardim do Tabaco, n°34, 1149-041 Lisbon, Portugal; Department of Clinical Neurosciences and Mental Health, Faculty of Medicine, University of Porto, Alameda Prof. Hernani Monteiro, 4200-319 Porto, Portugal; Anaesthesiology, Faculty of Medicine, University of Porto, Alameda Prof. Hernani Monteiro, 4200-319 Porto, Portugal

**Keywords:** Attachment, Cortisol, Pregnancy

## Abstract

**Background:**

Recent research has documented the association between attachment and cortisol rhythms. During pregnancy, when attachment patterns are likely to be activated, elevated levels of cortisol are associated with negative effects for the mother and the foetus. The aim of the present study was to examine the association of adult attachment style and cortisol rhythms in pregnant women.

**Methods:**

Eighty women in the third trimester of pregnancy participated in the study. Adult attachment was assessed using the Adult Attachment Scale – Revised (AAS-R). Participants collected 4 samples of salivary cortisol at two different days; 3 samples were collected in the morning immediately after wakeup and one sample was collected by bedtime.

**Results:**

Results found group significant differences in the cortisol diurnal oscillation (*F*_(1,71)_ = 26.46, *p* < .001,), with secure women reporting a steep decrease in cortisol from awakening to bedtime, while women with fearful avoidant attachment reported no changes. No group differences were found regarding the cortisol awakening response.

**Conclusions:**

These results highlight the importance of considering attachment patterns during pregnancy, suggesting fearful avoidant attachment style as a possible risk factor for emotional difficulties and dysregulation of the neuroendocrine rhythms.

## Background

In the last years, research is been increasingly concerned in understanding psychobiological processes through which emotional (de)regulation may affect distress and health outcomes. It has been shown that acute and chronic responses to stress may affect health outcomes in several conditions, namely through the activation of stress-response mechanisms, specifically the hypothalamic–pituitary–adrenal (HPA) axis [75]. The Attachment Theory [7] has a sound framework for explaining this association [67], as it claims that the attachment system is activated in stressful situations, being responsible for regulating the emotional and physiological responses to stress. Pregnancy is a stressful naturally occurring event that challenges the individuals’ wellbeing and is thought to activate attachment relations [79]. Although previous studies have focused on the HPA rhythm during pregnancy [54], there is no research on the effect of mothers’ attachment style on physiological response during pregnancy. A deeper understanding on these processes is of critical value because HPA deregulation may negatively affect the mother health and the fetus development [30].

According to attachment theory, human beings are equipped with an innate psychobiological system, the attachment behavioral system, accounting for a stable propensity of an individual to establish an emotional bond to others (attachment figures) for safety and security [7]. This system develops during infancy and early childhood based upon the interactions with primary caregivers, forming internal working models that are responsible for basic regulatory functions, particularly affect regulation and the management of stress-inducing events [7, 71], promoting an adaptive response to threat. Research has shown that attachment styles are associated with different stress responses and psychophysiological profiles [57]. When activated by a stressor, attachment system is activated and affects the way the individuals respond to the threat. Individuals with non-secure attachment, namely high attachment anxiety, tend to report high reactivity to threat, which is linked with higher HPA activity [65].

The attachment relationship is an interactive process marked by a strong mutual stimulation between the child and his caregiver [7], which is implicated in the circadian organization and responsiveness of the HPA axis [77]. Indeed, the early care provided by the mother is a significant predictor of the quality of a child’s attachment throughout life [81]. Maternal care also acts as a powerful *zeitgeber* (external cue for synchronization of biological rhythms) during the early periods of individual development and is essential for the synchronization of a child’s biochronometric system [77].

The relationship between the mechanisms that establish the link between attachment and the HPA axis are have even been supported by neuroimaging and neurophysiology [24], in which are shown, associations between limbic activity and cortisol responses [61, 63], the relationship between cortisol hypersecretion, self-esteem and hippocampal atrophy [60] and the connection between the dimensions of attachment and cell density of the hippocampus [64]. These data are consistent with the fact that glucocorticoids may disturb the neuronal plasticity [32], especially in the brain structures with a high density of receptors for glucocorticoids and characterized by prolonged postnatal developmental stages, as in the hippocampus which is particularly more susceptible to disturbances [74].

Circadian rhythms are the basis of anticipatory adaptation to environmental challenges [52]. During this adaptation, cortisol plays a key role in the internal synchronization of other body circadian rhythms [5, 23]. Cortisol is a biomarker of stress, and changes in its secretion are implicated in multiple diseases and disorders and are related to socio-economic, demographic and psychological factors [46–48, 72]. Examining the diurnal cycle of cortisol levels may clarify the influence of psycho-emotional factors on the HPA axis. Cortisol has a well-known circadian cycle, described by a rise in the morning followed by a steady decrease throughout the day, falling to low levels by midnight. According to Stone et al. [73], modifications of the circadian rhythm can be suggestive of dysregulation of the HPA.

Another cortisol rhythm has been described and exhaustively studied in recent years, namely, the cortisol awakening response (CAR) [16]. This is a distinct rhythm superimposed on the circadian oscillation, representing its acrophase and it primarily reflects the psychophysiological processes of the sleep-wake transition and may be linked to hippocampal preparation of the HPA axis to face an anticipated challenge [15]. The CAR is characterized by a marked increase in cortisol levels during the 45 min after waking [16, 29, 78]. The CAR patterns are related to multiple psychological and psychosocial factors [14] and discrepancies in results have been described in the literature. Therefore, it is important to note that a large set of confounders (e.g. gender, age, smoking habits, awakening time, day of measurement) [16] may affect the associations between CAR patterns and biopsychosocial measures.

There has been some research on the association between attachment and cortisol responses. Some studies have examined the effect of attachment relationships on cortisol levels in participants undergoing a stressful task [25, 37, 59], usually assessing salivary cortisol before and after the procedure. Despite the relevance of this reactive approach, when studying the regulation of HPA activation, it is also important to consider cortisol circadian rhythm, namely, its diurnal variation and the CAR. Regarding the diurnal variation in cortisol, a study by Adam and Gunnar [1] found that individuals with more positive relationships (conceptualized as securely attached women) reported more variant diurnal cortisol profiles (higher morning values and steeper diurnal cortisol slopes). Another study by Quirin et al. [65] found that higher cortisol responses to awakening were associated with lower attachment-related anxiety. A recent study [38] integrated both the diurnal variation and the dynamic increase in an investigation of the association between attachment style and cortisol responses in older adults. These authors found that the adult attachment was associated with a diurnal cortisol pattern, with preoccupied attachment ratings associated with a flatter cortisol profile across the day due to heightened bedtime cortisol levels. In this study, the CAR was not associated with attachment. In conclusion, research suggests that there is an association between attachment and cortisol patterns. Some apparent inconsistencies may be explained by different research protocols (natural stress vs. stress following laboratory procedures) [53] or the occurrence of multiple confounders (e.g. age, gender) [16].

Although several studies on attachment relationships and physiological outcomes have been conducted, to the authors’ knowledge, there has been no research focusing on the association of attachment with cortisol responses during pregnancy. Pregnancy is a significant life transition that requires adaptive efforts in several domains of the parents’ lives to deal with the challenges of the transition to parenthood [19]. According to attachment theory, as a naturally occurring stressful event, pregnancy should activate the attachment system to elicit a complex interplay of cognitions, emotions, and behaviors, in order to increase proximity to attachment figures [79]. In addition, pregnancy is particularly connected to attachment relationships [28], as it is activates relations with the partner, family of origin and future child [51]. Both attachment and cortisol rhythms are stable internal resources that can affect the psychological and biological adaptations to pregnancy, and maladaptation can lead to HPA dysregulation and therefore negatively affect maternal and fetal health [30, 35].

Considering the importance of these systems to both the woman and the child and taking into account the possible convergence of the activation of stable internal resources (behavioral and neuroendocrine) with the adaptive responses to pregnancy-related challenges, this study aimed to assess cortisol responses in pregnant women in the third trimester of pregnancy and to examine the association between attachment style and cortisol secretion, specifically cortisol diurnal rhythm and the CAR.

## Methods

### Participants and procedures

This cross sectional study was carried out in compliance with the Helsinki Declaration. Ethical approval was obtained from the Ethics Committee of the Maternity Hospital Doctor Alfredo da Costa (Lisbon, Portugal) and from the National Commission of Data Protection. All participants signed a written informed consent, which included the right of withdrawing from the study at any point without compromising their clinical treatment. The participants received no compensation for their participation in the study.

The sample collection took place between April 2010 and September 2011. A combined convenience and consecutive sampling approach was used. Women were recruited in the general obstetrics appointment of the maternity department on the basis of the researchers’ convenience. The inclusion criteria for participants in this study were as follows: pregnant women above 18 years old; healthy, singleton pregnancy; nulliparous or parous (up to a third pregnancy); absence of psychopathological disorder and substance abuse; absence of medication and of biological disease affecting the adrenal function.

The patients were invited to participate in the study in their third trimester of pregnancy (26 weeks or later). Data were obtained regarding their sociodemographic and obstetric factors, and the participants completed the Adult Attachment Scale – Revised (AAS-R). A total of 132 pregnant women agreed to participate in the study, but 52 participants (39.4 %) were excluded from the study. Motives for this exclusion were incorrect completion of the AAS-R (*n* = 5), incomplete demographic and clinical data (*n* = 5) and lack of reliability in the salivary cortisol assessment (*n* = 42). Therefore, the final sample of this study consisted of 80 pregnant women. Participants that were excluded from the study for incomplete demographic and clinical data and low reliability of salivary cortisol assessment did not differ from the participants in most demographic, health status or obstetrical data, although there were more parous women in the former group (70.2. % vs. 48.8 %, *χ*^2^ = 5.55, *p* = .018). Differences were found in the attachment-related anxiety scale, with women from the excluded group (M = 2.24, DP = .74) reporting lower levels of attachment anxiety than women in the final sample (M = 2.65, DP = .83) (*t*_125_ = 2.760, *p* = .007).

### Measures

Sociodemographic data (age, marital status, and educational level), clinical and obstetric data (parity, pre-pregnancy Body mass index (BMI) and gestational time) were collected by interview by the main researcher, who was also part of the clinical staff of the maternity hospital. The gestational ages were recorded again at the time of sample collection.

### Assessment of salivary cortisol

Data collection protocol: The participants were asked to collect saliva samples using synthetic swab Salivettes (Sarstedt - Code Blau®, Ref. 51.1534.500. Germany) at four time points: immediately after awakening (S1), 15 min (S2) and 45 min later (S3) and at bedtime, between 11 pm and 12 pm (S4). The participants were instructed to collect the samples on two different days and to record the hour of awakening. Each participant received a collection kit with eight salivettes (each salivette was coded and numbered for the sequence of saliva samples) and a detailed instruction sheet.

In the interview, all the collection procedures were discussed, the use of the salivettes was demonstrated, and the participant's questions were addressed. The instruction sheet detailed the following saliva collection process: the collection should be identical on both days; for the first three samples, participants should not brush their teeth before saliva collection (to avoid micro-injuries in the oral cavity that could cause blood contamination of the sample); should not eat (although the fasting period should not exceed 12 hours) or take any medication before the saliva collection; and, in the fourth sample, participants should refrain from eating or taking any medication in the 45 min prior to saliva collection. The participants stored the tubes in their refrigerators and returned the salivettes to the researchers during the next medical appointment at the maternity hospital. To ensure the stability of the saliva cortisol, the samples were stored frozen at −20° until analysis for a period no longer than nine months. The cortisol concentrations were measured by Enzyme-Linked-Immunosorbent-Assay (ELISA) (DIAsource ImmunoAssays ©, Belgium). This method presented a cross reactivity below 7,6 % with other steroids present in the saliva. Intra-and inter-assay coefficients of variance were less than 10,3 % and 9,8 %, respectively. Analysis of the measurements required a calibration curve and duplicate controls. Only the samples with correlation coefficients between that of the duplicate and the validated controls were considered. The value of each sample was calculated by averaging two measurements.

Compliance with the protocol: During participant training, the importance of rigor and honesty in the saliva collection and the data recording was emphasized. The time of awakening and saliva sampling times were self-reported. Following the recommendations of Kunz-Ebrecht et al. [41]), samples revealing protocol noncompliance (a reported deviation from the scheduled time for the first three samples greater than 10 min) were excluded from the analysis.

### Adult attachment

Adult attachment was assessed using the Portuguese version of the Adult Attachment Scale – Revised [13]. The AAS-R [18] consists of 18 items scored on a 5-point scale ranging from 1 (Not at all characteristic of me) to 5 (Extremely characteristic of me), organized in three subscales: Anxiety, Comfort with closeness and comfort with depending on others. According to Brennan et al. [8]) these subscales are further organized in two dimensions: attachment anxiety and attachment avoidance. Individuals who score highly on the attachment anxiety tend to display an excessive concern with their own distress and negative emotions and to overreact to their negative feelings in order to elicit support from others. Individuals who score highly on attachment avoidance tend to seek distance cognitive and behavioral) from the stressful event, seeming less sensitive to it, and avoid seeking emotional or instrumental support from others [44, 50]. Higher scores are indicative of more anxious and/or avoidant working models (i.e., insecure working models). In this sample, the reliability values were .87 (Avoidance) and .89 (Anxiety). For comparison analyses of the attachment profiles, the participants were assigned to their respective attachment styles based on whether their scores on the attachment-related anxiety and avoidance dimensions were above or below the scale midpoint (3).

### Data analysis

Cortisol data analysis protocol: Considering the study aims, both circadian rhythm (diurnal response) and the CAR were assessed. The circadian rhythm of cortisol secretion was assessed by computing the difference score of the first S1 (cortisol at awakening) and last S4 (cortisol by bedtime) samples of the day. The CAR was assessed using the first three samples of the day: at awakening (S1), + 15 min (S2) and + 45 min (S3). To measure the CAR, we followed the approach proposed by Clow et al. [15]. The S1 and the area under the curve with respect to the increase in cortisol concentration (AUCi) provide two clearly distinguishable measures. These values clarify differences in the end state of the pre-awakening cortisol secretion (S1) or in the post-awakening response (the dynamic increase, calculated by the AUCi). The AUCi was calculated following the recommendations of Pruessner et al. [62]) The hour of awakening was calculated as the total minutes between midnight and the hour that was reported by the participants as the time of awakening.

Statistical analysis: Data analyses were conducted with IBM SPSS, version 20.0. Desciptive statistics with means and standard deviations (*SD*) were reported for continuous variables and frequencies for categorical variables. Pearson correlations were used to examine the associations between the study variables. Paired-sample *t* tests were used for comparing differences in the hormonal output between the two days. Cortisol levels across different time points were analyzed with analysis of variance using the General Linear Model (GLM) for repeated measures, using the time of the measurements as a within-subjects factor and controlling for the effect of age, BMI and gestational week. When the assumption of sphericity was violated, the Greenhouse-Geisser correction was applied. When testing the differences of cortisol measurements among group of participants, a between-subject factor was included (e.g. Attachment style: 0 = secure; 1 = fearful avoidant). For this analysis, based on Cohen’s recommendations [17] for a significance level of .05 and a power of .80, this sample size provides adequate statistical power for detecting small effects (*f* = .15) [26].

## Results

### Participants’ characteristics

The sample consisted of 80 women in the third trimester of pregnancy (median = 33 weeks). All participants were married or cohabiting and the majority was nulliparous. When computing attachment styles, few women were identified as having dismissing (N = 6, 7.4 %) and fearful/preoccupied attachment styles (N = 3, 3.7 %). Therefore, these participants were excluded from the following analyses. The participants’ characteristics are presented in Table 1. All the participants were women of reproductive age who exercised little and reported no medication, caffeine or alcohol consumption.

**Table 1:** Demographic and obstetrical-gynaecological characteristics by attachment style

	Total (*n* = 71)	Secure (*n* = 42)	Fearful avoidant (*n* = 29)		
	Mean ± SD (Min-Max)	Mean ± SD (Min-Max)	Mean ± SD (Min-Max)	*t*_(79)_	Cohen’s *d*
Age (years)	32.17 *±* 4.98 (20–45)	31.21 *±* 4.76 (20–40)	33.55 *±* 5.04 (24–45)	1.99	0.48
BMI pre-pregnancy	24.56 ± 3.90 (16–38)	24.88 ± 4.17 (16–38)	24.09 ± 3.49 (18–32)	−0.84	0.21
Gestation week	33.01 ± 3.55 (27–40)	33.02 ± 3.25 (27–40)	33.00 ± 3.95 (28–40)	0.03	0.01
	*n* (%)	*n* (%)	*n* (%)	*χ* ^2^	Cramer’s *V*
Education				0.51	0.084
Basic (9 years)	12 (16.9)	6 (14.3)	6 (20.7)		
Secondary (12 years)	26 (36.6)	16 (38.1)	10 (34.5)		
University	33 (46.5)	20 (47.6)	13 (44.8)		
Parity				0.12	0.040
Nulliparous	36 (50. 7)	22 (52.4)	14 (48.3)		
Parous	35 (49.3)	20 (47.6)	15 (51.7)		

No statistically significant differences were found between the two groups in any of the reported variables; BMI pre-pregnancy was kg/m^2^;

### Circadian rhythm and CAR in pregnant women in the third trimester of pregnancy

Cortisol levels are presented in Table 2. The cortisol levels for the two assessment days showed high stability, with Pearson correlations among the measures for the two days for each time point ranging from .58 to .70 (*p* < .001). The differences between these measures were not significant (*p* > .05). Therefore, for further analysis, the cortisol values for each time point were averaged across the two days.

**Table 2:** Descriptives (Mean and standard deviations) of cortisol measurements in the four time points

	Mean	SD	Range (Min-Max)
At awakening (S1)	12.78	5.42	2.84–30.95
+15 min (S2)	15.72	6.05	3.95–36.64
+45 min (S3)	16.66	6.88	5.16–36.90
Bedtime (S4)	10.51	6.09	2.15–32.38

Cortisol values are expressed in nmol/l

Throughout the day, the participants exhibited a significant decrease between S1 and S4 (*t*_(71)_ = 4.60, *p* < .001, Cohen’s *d* = .55), suggesting that the circadian rhythm was maintained in normal pregnancy. Diurnal variation was not related to the women’s age, gestational age, pre-pregnancy BMI or time of awakening (all *p* > .05).

Regarding the CAR, a significant increase was found in cortisol concentrations along the three measurement points (*F*_(2,69)_ = 36.67, *p* < .001, *η*_*p*_^2^ = .50). Within the first 15 min, cortisol levels increased approximately 23 %; in the following 30 min, the levels increased approximately 6 %. These results suggest that the CAR was maintained in normal pregnancy.

Table 3 presents the descriptive statistics and intercorrelations for the cortisol measures, the aggregated measures and the awakening hour. Cortisol at awakening (S1) and AUCi were not significantly associated with age, gestational age, pre-pregnancy BMI, parity or the time of awakening (*p* > .05 for all factors). Similarly, cortisol at awakening was not associated with the AUCi. The AUCi was also negatively associated with the cortisol circadian rhythm; that is, a smaller decrease from awakening to bedtime was associated with a higher dynamic increase in the CAR.

**Table 3:** Means, standard deviations, and intercorrelations for the study variables

Variables	Attachment –Anxiety	Attachment –Avoidance	Cortisol S1	Diurnal cortisol	AUCi
Attachment – Anxiety	-				
Attachment – Avoidance	.83***	-			
Cortisol S1	.10	.08	-		
Diurnal cortisol (S1 – S4)	-.41***	-.46***	.21	-	
AUCi	-.03	-.09	-.07	-.43***	-
Age	.29*	.15	-.16	-.05	-.07
Gestational age	-.01	.04	.06	-.09	.09
BMI pre-pregnancy	-.07	-.06	-.13	-.04	-.04
Parity	-.05	.08	.08	-.01	-.03
Mean	2.68	2.86	12.78	2.27	124.51
SD	0.86	0.63	5.642	4.15	125.48
Observed range (min – max)	1 – 4.17	1.58 – 3.92	2.84 – 33.95	−9.53 – 10.	−134.74 – 565.71

*BMI*, Body Mass Index; *AUCi*, Area under the curve regarding the increase for cortisol awakening response (CAR); Parity: 0 = Nulliparous; 1 = Parous

*p < 0.05; **p < 0.01; ***p < 0.001

### Attachment styles and cortisol responses

A range of preliminary analyses was conducted to examine the associations between cortisol secretion and attachment dimensions as well as demographic and clinical variables (Table 3). The results showed that attachment-related avoidance and anxiety were negatively correlated with the cortisol circadian rhythm, that is, higher attachment anxiety (*r* = −.41, *p* < .05) and avoidance (*r* = −.46, *p* < .05) in pregnant women were associated with lower diurnal cortisol variation. Additionally, age was positively correlated with attachment anxiety (*r* = .29, *p* = .015).

#### Cortisol circadian rhythm in secure and fearful avoidant attachment

**Fig. 1:**
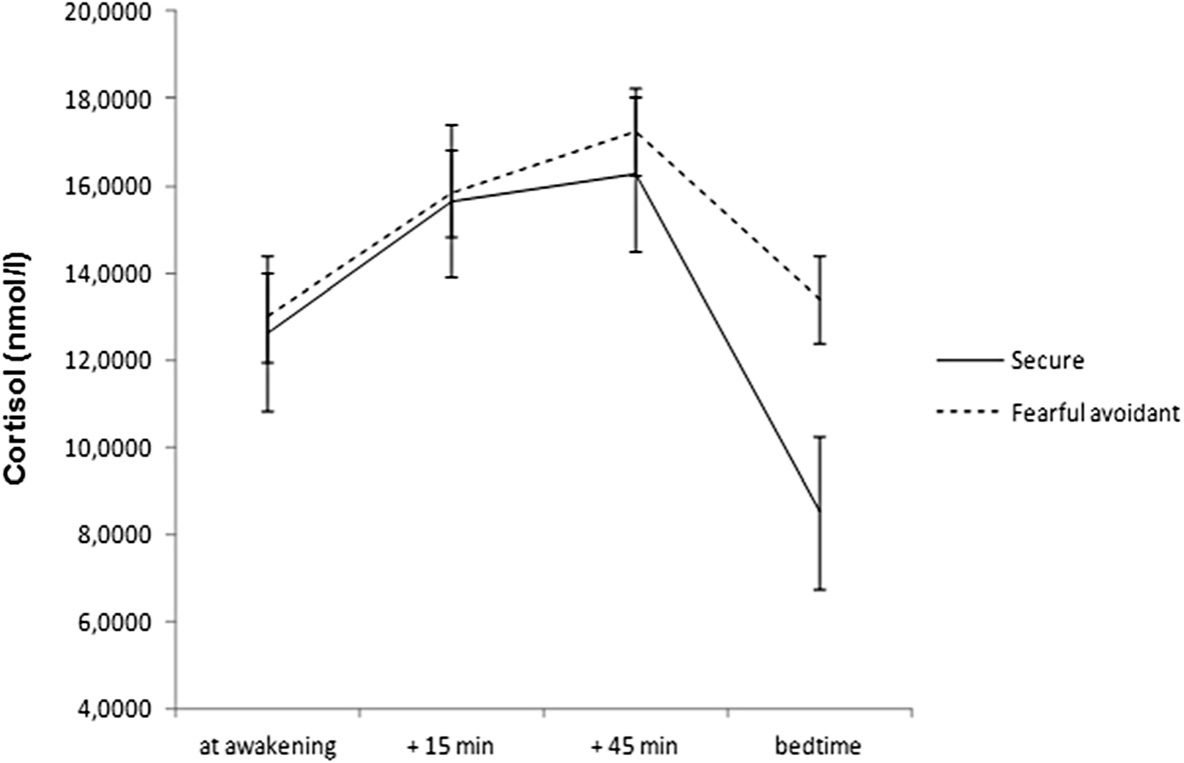
Cortisol responses in securely and insecurely attached pregnant women at awakening, +15 min, + 45 min and by bedtime

Regarding the cortisol circadian rhythm, differences were found between secure and fearful avoidant attachment women (*F*_(1,71)_ = 26.46, *p* < .001, *η*_p_^2^ = .29). Further analysis revealed that securely attached pregnant women reported a sharp decrease from S1 to S4 (*t*_(41)_ = 10.88, *p* < .001, Cohen’s *d* = 1.68), while no differences were found from morning cortisol to bedtime cortisol in women with fearful avoidant attachment (*t*_(28)_ = −0.48, *p* = .637, Cohen’s *d* = .08). When exploring differences on the diurnal profile, results demonstrated that group differences relied on the pre-bed cortisol levels (*t*_(69)_ = 3.598, *p* = .002, Cohen’s *d* = 0.69), as detailed in Fig. 1.

#### Cortisol Awakening Response in secure and fearful avoidant attachment

The CAR, as measured by cortisol secretion at S1 and AUCi, was not significantly associated with any of the studied dimensions of adult attachment (attachment-related anxiety or avoidance). No significant differences were found regarding cortisol at awakening (S1) (*t*_*69*_ = 0.27, *p* = .798, Cohen’s *d* = .06) or AUCi (*t*_(69)_ = 0.14, *p* = .89, Cohen’s *d* = .03) between the two groups.

## Discussion

The present study examined the association between attachment styles and cortisol secretion in pregnant women. Although several studies have examined the association between adult attachment and cortisol responses, to date no studies have focused on this association during pregnancy. Considering the link between the attachment system and neurohormonal responses to stress [7, 75], a deeper knowledge of the association of attachment with cortisol response in pregnancy is important, as it may allow for the identification of risk factors for the mother’s and the child’s biopsychological well-being [10, 28, 30, 35, 45, 49]. Specifically for the latter it may be of foremost importance, due to the consequences of this dysregulation which can be established since the intrauterine environment throughout the individual’s development the first years of life [66]. Indeed, as the exposure of the foetus to excess glucocorticoid may result in intrauterine growth failure and affects the foetus health in adult life, namely physical and mental development, temperament and cognitive performance [56, 58, 68].

Regarding our first objective, which was to study cortisol responses in pregnant women in the third trimester of gestation, the results showed that the cortisol circadian rhythm and the CAR were maintained in normal pregnancy. This outcome is in accordance with previous research and is expected in a normal population [3, 22]. The absence of significant associations with sociodemographic (e.g., age) and clinical variables (e.g., gestational age) with cortisol, as opposed to other research findings [4, 11, 76], may be due to the homogeneity of this study sample (limited age frame, inclusion of pregnant women in the third trimester of pregnancy).

A negative correlation between circadian rhythm and dynamic variation post awakening (AUCi) was found, confirming the association of a flatter profile and an accentuated CAR. This result suggests that the disruption on this cortisol basal rhythm [73] consequence of the elevated cortisol levels by bedtime may be related with the dynamic of the awakening response. Indeed, previous research has documented the association between the CAR and psychological outcomes, such as anxiety, depression or work stress [14, 31, 41, 42, 80] or during pregnancy, as related with fear regarding the anticipation of childbirth [2]. Therefore, CAR is very likely to be sensitive to chronic stress and its anticipation [41]. In addition, high evening cortisol levels are associated with stress [21], which can explain this study result namely considering the pregnancy as a stressful event both at a biological and psychological level.

Regarding our second objective, the results highlight the negative correlation between attachment anxiety and avoidance and decreased circadian cortisol oscillation, illuminating the associations between attachment and cortisol response. The most significant results of this study show that securely attached women reported an expected circadian rhythm, with elevated cortisol levels in the morning and a decrease in night levels, while women with fearful avoidant attachment showed a flatter cortisol profile, with no significant differences from morning to evening, which suggests a disruption of this rhythm. Cortisol presents a marked circadian rhythm that depends on the central pacemaker, located in the suprachiasmatic nucleus of the hypothalamus. The neurohormone acts as a secondary messenger in the real interaction between this central pacemaker and the peripheral pacemakers [23], promoting delays or advances in the latter [5] and making their circadian rhythm essential to the internal synchronization of other circadian rhythms in the body. The cortisol pattern found in the current study may be due to a hypervigilant strategy used by fearful avoidant attachment women during the day that may prevent them from “turning off” and reducing the levels of activation as bedtime approaches. Insecurely attached women are similarly activated in the morning and at bedtime, contrary to the expected cortisol profile. This result is relevant and is partially in line with the findings by Kidd et al. [38], who found that participants with elevated attachment anxiety (preocupied attachment) was associated with a flatter cortisol profile over the day. The authors concluded that these results are derived from heightened cortisol output at bedtime, as we found in our study, which is possibly due to high elevels of attachment anxiety. Notwithstanding, it is important to mention that the absence of amplitude of the diurnal profile in the fearful attachment group in our study results is particularly relevant as it concerns pregnant women, for whom, albeit the pregnancy hypercortisolism [43, 55], is expected an attenuation of the endocrine response [69, 70].

The examination of the association between the CAR and attachment dimensions revealed no significant effects. Previous research on the significance of the CAR responses has yielded different findings [14], with some research relating a disruption of the CAR to attachment patterns [65], while others failed to support this association [38]. More specifically, no significant association was revealed between the CAR and the occurrence of mood disorders in pregnancy [33]. That the CAR is highly vulnerable to the influence of situational factors [14] may explain, in part, the failure in this study to achieve a significant finding. Future research using larger samples and assessing other situational and psychological factors may be helpful to clarify the association of the CAR and attachment during pregnancy.

### Limitations

Some limitations should be considered when interpreting our findings. First, sample recruitment was based on non probabilistic methods, which does not warrant that the sample is representative of the population of pregnant women and therefore limits the generalizability of our findings. The use of random selection methods of participants would overcome this limitation and should be considered in future studies. In addition, a large number of participants were excluded for the final sample, mainly due to noncompliance with the study protocol, and the excluded group differed significantly from the final sample, as the former was most likely to be multiparous and to report lower attachment anxiety. The lower compliance rate of multiparous women may be due to the difficulty in complying with the collection of saliva samples in the morning (at awakening and 45 minutes later) when having another child to take care of [12], although parity is not expected to affect cortisol output [27]. Higher scores on attachment anxiety on the final sample, compared to women in the excluded group, may be explained by the high tendency to value proximity and support of others, including the medical team, increasing the chance of participating in the study, and may have affected the constitution of the sample. Therefore, the proportion of women in the final sample may not be representative of the pregnant population.

Second, our sample did not allow for the analysis of cortisol output using the four categories of attachment style, as proposed by Bartholomew and Horowitz [6], due to the small number of participants in the preoccupied and dismissing styles. For this reason, participants from these groups were not included in the analysis as it may have limited the interpretation of the results as different cortisol profiles could have been found. Indeed, it would have been important to note whether different profiles of anxiety and avoidance, as different tendencies to respond to threat and stress, were more likely to be associated with hyper activation (more likely in elevated attachment anxiety) or deactivation (more likely in elevated attachment avoidance) of the cortisol profiles. Further studies on larger samples that include participants in the four categories may indicate more specific associations between each of the styles and cortisol responses. Additionally, attachment was assessed using a self-report measure. Since attachment reflects the individual’s subjective perceptions of their close relationships, it is likely that participants may be vulnerable to reporting bias. Replication of this study with other methods of data collection, such as the Adult Attachment Interview [34] would strengthen the validity of the findings reported herein. The assessment of other psychological constructs would also have been important. In future studies, this is an important requirement, in order to account for possible psychological confounders, such as anxiety, depression, stress or other psychosocial factors that can influence cortisol output in the day of sample collection, or other variables such as individual characteristics (e.g., personality traits such as neuroticism) and social support that can affect the association between attachment and cortisol secretion. Furthermore, the maternal-foetal attachment, defined as the affiliation and interaction behaviours towards the foetus [20], may be influenced by attachment representations and affect the emotional adaptation to pregnancy and consequently psychophysiological responses, and therefore should also be considered in future studies. The use of just two cortisol measures to compute the diurnal profile of cortisol responses may limit the interpretation regarding the circadian cortisol rhythm. However, Kramer et al. showed that slopes based two daily time points are correlated highly with those based on four points [39]. Finally, the use of self reports for recording the hour of collection may be subject to bias, and some author recommend the use of electronic monitoring [9, 36, 40]. However, according to previous research, self-reports are highly correlated with electronic monitoring [4, 39].

## Conclusions

This is the first study providing evidence for an association between insecure attachment and basal circadian changes, assessed by measuring the oscillation of cortisol in the last trimester of pregnancy in pregnant women free of obstetric pathology. The findings reported herein emphasize the congruence between behavioral and neuroendocrine internal resources in coping with stressful events and the stability of these resources through life. These changes can have important consequences on the mother’s and the child’s physical and psychological health. These findings can contribute to the development of an innovative and prophylactic health care strategy focused on the experience of pregnancy and the transition to parenthood, aimed at promoting healthy development for both mother and child. Despite the well-known methodological limitations of naturalistic research, this study also highlighted the importance of conducting research in real life contexts, allowing for the study of psychological and neuroendocrine responses to naturally-occurring life events.

Although the study has important contributions, the design was cross-sectional and, therefore, no causal interpretations can be drawn. In addition, because other psychological constructs that could influence the association between attachment style and cortisol were not examined, the results should be interpreted with caution.

In conclusion, our study clarified the association between attachment style and cortisol profile during pregnancy. Securely attached women reported an expected circadian rhythm, with elevated cortisol levels in the morning and a decrease in night levels. However, women with fearful avoidant attachment showed a flatter cortisol profile, with no significant differences from morning to evening, which suggests a disruption of the cortisol rhythm from morning to evening. These results highlight, therefore, the association between non secure attachment in and the primary mechanisms of adaptation to the environment.

## Abbreviations

HPA: hypothalamic–pituitary–adrenal
CAR: Cortisol awakening response
AUCi: Area under the curve
BMI: Body Mass Index
